# Medication changes after switching from CONCERTA® brand methylphenidate HCl to a generic long-acting formulation: A retrospective database study

**DOI:** 10.1371/journal.pone.0193453

**Published:** 2018-02-28

**Authors:** Daniel Fife, M. Soledad Cepeda, Alan Baseman, Henry Richards, Peter Hu, H. Lynn Starr, Anthony G. Sena

**Affiliations:** 1 Epidemiology, Janssen Research & Development, LLC, Titusville, NJ, United States of America; 2 Global Medical Safety, Janssen Research & Development, LLC, Horsham, PA, United States of America; 3 Established Products, Janssen Research & Development, LLC, Titusville, NJ, United States of America; 4 Clinical Biostatistics, Janssen Research & Development, LLC, Raritan, NJ, United States of America; 5 Janssen Scientific Affairs, LLC, Titusville, NJ, United States of America; University of North Carolina at Chapel Hill, UNITED STATES

## Abstract

**Background:**

Observational studies of switching from branded to generic formulations of the same drug substance often lack appropriate comparators for the subjects who switched. Three generic formulations were deemed equivalent to Concerta: an authorized generic (AG) identical except for external packaging, and two other generics (EG).

**Objective:**

Compare the incidence of a combined endpoint (switching back to Concerta, changing the use of immediate release methylphenidate (MPH), stopping all long-acting methylphenidate, or starting a new medication) among people switched from Concerta to the AG versus the EG.

**Methods:**

Cohort study from the Truven CCAE database of people aged 6 to 65 diagnosed with ADHD, treated with Concerta, and switched to the EG or to the AG formulation.

**Results:**

In the EG arm 24.6% and in the AG arm 19.7% of subjects switched back to Concerta. The proportion of subjects meeting the combined endpoint was 39.5% in the EG arm, 32.9% in the AG arm, a crude risk ratio of 1.20 (95% CI 0.94, 1.54). After adjustment by propensity score stratification, the adjusted odds ratio (OR) was 1.23 (95% CI 0.90, 1.70). In an unplanned analysis using a different method of adjustment, the adjusted OR was 1.00 (95% CI 0.69, 1.44).

**Discussion:**

This study did not detect a difference between the proportion of people who met the study endpoint in the two study arms, i.e. between those who switched to a generic formulation that was identical to Concerta except for external packaging and those who switched to the comparison generics. The high incidence of the combined endpoint in the AG arm demonstrates the need for an appropriate comparator in studies of this type.

**Trial registration:**

ClinicalTrials.gov NCT02730572

## Introduction

The need to control health care costs has led to substantial numbers of patients being switched from branded formulations to generic versions of the same or similar active drug substances, and many authors have sought to use retrospective analyses of health services databases to investigate the clinical consequences of such substitution. These studies typically focus on how often some measure of failure of the new medication occurred among patients who made the substitution. Commonly used measures of failure include changes in requirements for care, changes in the clinical status of the condition the medicine treats, or return to the branded product. As distinct from prospective clinical studies that can set up an appropriate comparator [1], retrospective studies must make do with the comparators that are available, such as the frequency of the measure of failure among the same patients prior to the substitution, or among other patients who did not experience the substitution. In retrospective studies from health services databases, the options for a measure of failure are also restricted by the fact that such databases offer information about diagnoses and treatments, but little or no direct information about clinical status so in such studies the measure of failure must be constructed from diagnoses and treatments.

Examples of such database studies of generic substitution include: Wu [1] who found that among adult patients with major depressive disorder treated with a branded SSRI, those who were switched to a generic SSRI for nonmedical reasons had more mental health hospitalizations and emergency department visits during the 6 months after their switch than did age-and-index-date matched subjects with major depressive disorder treated with a branded SSRI who were not switched to a generic SSRI. Cheetham [2] found that lipid profiles improved slightly after patients were switched from branded simvastatin to generic lovastatin. Erickson [3] who compared patients who switched from branded to generic formulations of three antiepileptic drugs (AEDs) to propensity score matched patients who remained on the branded formulations and found no difference in all-cause emergency department visits or hospitalizations but, for one of the three drugs found a nearly two-fold difference in a composite endpoint of discontinuation of the index AED, change in dose of the index AED, or addition of another AED. Andermann [4] who found that after being switched from branded formulations to generic formulations of three antiepileptic drugs, 12% to 20% of subjects switched back to the branded formulation, and among those who remained on one of the generic drugs, the daily dose increased after the substitution. In contrast, the same study found that 1.5% of subjects who were using a branded formulation of a statin and 2% to 3% of subjects who were using branded formulations of selective serotonin reuptake inhibitors switched back to the branded formulation after being switched to generic formulations.

Comparisons of people who were switched from branded to generic formulations versus the past experience of the same people, or versus the experience of other people who did not change formulations, have a potentially important limitation: The group that changed from the branded to the generic formulation was exposed to the effects of any pharmacologic differences between the two formulations and also to any non-pharmacological effects, e.g., the psychological effects of switching from a familiar and more expensive formulation to a new and less expensive one, but the comparators had neither of these exposures. Thus, any observed difference between the two groups could reflect a pharmacologic effect of switching, a non-pharmacologic effect of switching, or a mix of the two. Work on placebos, [5, 6] and on complaints of lack of effectiveness after a change in the label of an otherwise unchanged medication [7] demonstrate that the way a medication is presented may substantially influence the patient’s perception of its effectiveness. We sought to examine whether, in spite of the limitations described above (absence of direct information about clinical status, and the presence of psychological as well as pharmacological effects), it would be possible, through a retrospective study from a health services database, to detect a difference between measures of failure between subjects switched to the authorized generic formulation compared to other extended-release formulations of the same active drug substance that were deemed at the time to be therapeutically equivalent. A recent study of patients who were switched to authorized vs. other generics found similar hazard ratios for switching back to the branded formulation [8]and for outpatient visits, urgent care visits, hospitalizations, and medication discontinuation, and a slightly higher hazard ratio for emergency department visits among the group using authorized generics [9]. However, the generics Hansen studied did not subsequently lose their therapeutic equivalence status.

CONCERTA is a modified-release formulation (also described as extended-release or ER formulation) of methylphenidate HCl (MPH) manufactured by Janssen-Cilag Manufacturing, LLC for Janssen Pharmaceuticals, Inc. MPH is a central nervous system stimulant indicated for the treatment of attention deficit hyperactivity disorder (ADHD) in people aged 6 to 65 years. Beginning in May of 2011, an extended-release authorized generic formulation of MPH (termed in this study the Authorized Generic or AG formulation) was marketed in the United States by Actavis as a generic formulation equivalent to Concerta under agreement with Janssen. It is identical to Janssen’s Concerta except for the external packaging (the bottle that holds the tablets and the box that holds that bottle and the package insert) and the removal of the brand from the package insert. This formulation and two other extended-release generic MPH formulations (termed in this study the Equivalent Generic or EG formulations), one manufactured by Mallinckrodt Pharmaceuticals and approved by the Food and Drug Administration (FDA) in December 2012, the other manufactured by Kudco and approved by the FDA in July 2013, received therapeutic equivalence ratings of AB from the FDA (Orange Book, https://www.fda.gov/drugs/developmentapprovalprocess/ucm079068.htm, 2017). This rating allowed pharmacists to substitute the EG formulations for Concerta unless the prescription specifically called for no substitution. In 2015 Lally [10] reported a retrospective study of 14 ADHD patients who had been prescribed Concerta but received the EG formulations, did not show adequate improvement on their Inattention Scale [11], and were subsequently switched to Concerta with statistically significant improvement on that scale. Also In 2015, the FDA revoked the AB ratings of the latter two formulations and stated that “*An analysis of adverse event reports*, *an internal FDA re-examination of previously submitted data*, *and FDA laboratory tests of products manufactured by Mallinckrodt and Kudco have raised concerns that the products may not produce the same therapeutic benefits for some patients as the brand-name product*, *CONCERTA*, *manufactured by Janssen Pharmaceuticals*, *Inc*. *Janssen also manufactures an authorized Concerta generic*, *which is marketed by Actavis under a licensing agreement and is identical to Janssen’s CONCERTA*. *FDA included the authorized generic in its analysis and found it to be bioequivalent to, and substitutable for, CONCERTA. Apart from the Mallinckrodt, Kudco, and Actavis products, there are no other generics for CONCERTA*.” (http://www.fda.gov/Drugs/DrugSafety/ucm422568.htm, 2016). The fact that between 2012 and 2015 some patients switched from CONCERTA to the AG formulation and others switched from CONCERTA to the EG formulations, offered an opportunity to do a retrospective database study that would let us break the usually perfect correlation between the pharmacologic and non-pharmacologic effects of switching from branded to generic formulations of the active drug substance. We therefore compared a group of patients with ADHD who were treated with CONCERTA and switched to the AG formulation and thus were subject only to the non-pharmacologic effects of being switched vs. a group of patients who were treated with Concerta and were switched to the EG formulation and thus were subject to both the non-pharmacologic and the pharmacologic effects of being switched.

## Materials and methods

### Ethics statement

This work was based entirely on fully anonymized claims data. Apart from the claims data, no other information about patient records, and in particular no charts, were used. The New England IRB has indicated that such research is not considered human subjects research.

### Methods

Data for this study came from the Truven MarketScan Commercial Claims and Encounters (CCAE) database, in the Observational Medical Outcomes Partnership (OMOP) Common Data Model (CDM) version 5 format, from 2012, the year before the EG formulations became available, through January 31, 2015, the most recent date for which the data were available for analysis. CCAE is a US administrative health claims database with information on diagnoses, prescription medication dispensings, outpatient visits, and hospitalizations for active employees, early retirees, COBRA continues, and their dependents insured by employer-sponsored plans.

This was a retrospective cohort study whose subjects were prevalent users of Concerta. Subjects entered the study cohort when they met the following conditions: They were in the database continuously (except for breaks of <30 days) for at least 183 days after June 1, 2012; received a diagnosis of ADHD on or after January 1, 2012; used Concerta for at least 60 days continuously (meaning that each dispensing occurred within 15 days of the end of the days’ supply of the previous dispensing) and received a dispensing of the AG or EG formulation within 15 days of the end of the days of Concerta supplied. The date of that dispensing of the AG or EG formulation was the subject’s index date, and the AG or EG formulation was the subject’s exposure. The index date was required to be between Dec 1, 2012, the approximate date when the EG preparation became available, and Dec 3, 2014—to allow 60 days follow up by Jan 31, 2015, which is the end date for the available data. Subjects were required to be between age 6 and 65 on their index date. Subjects aged less than 21 years were required to have an index date between November 15 and April 15. This requirement was intended to provide 60 days before the substitution and 60 days after the substitution that did not overlap the period from June 15 to September 15, when many children and adolescents are out of school and may take a “vacation” from MPH. Subjects left the cohort with the first of: Meeting the primary endpoint; passage of 60 days since the subject’s index date; leaving the database (ignoring breaks of <30 days); receiving, after June 1, 2012, an excluded diagnosis; receiving, after their index date, a dispensing of a LA MPH other than Concerta or the LA MPH to which they were switched. Any subject who received EG and then AG, or vice-versa, or received both EG and AG formulations on the same date, left the cohort when that occurred. No subject was permitted to enter the study cohort more than once.

The excluded diagnoses corresponded to conditions that were the subject of warnings or contraindications in the US label for Concerta: Renal or hepatic insufficiency, schizophrenia, bipolar disorder or mania, anxiety, glaucoma, Tourette’s syndrome, nervous tension (ICD-9 codes 799.21, 300.9), or narrowing of esophagus, stomach or intestine. Subjects were also excluded if: Their age or sex was not specified; between 183 days before joining the cohort and 60 days after their index date they were diagnosed as pregnant or were dispensed an antidepressant or antipsychotic or a medication commonly used to treat seizures or migraines; between 60 days before their index date and 60 days after their index date they were dispensed MPH in a form other than a non-chewable tablet, or were dispensed a LA MPH other than Concerta, the AG MPH formulation or the EG MPH formulation; or they received a dispensing of Concerta within 3 days after their index date.

**Fig 1:**
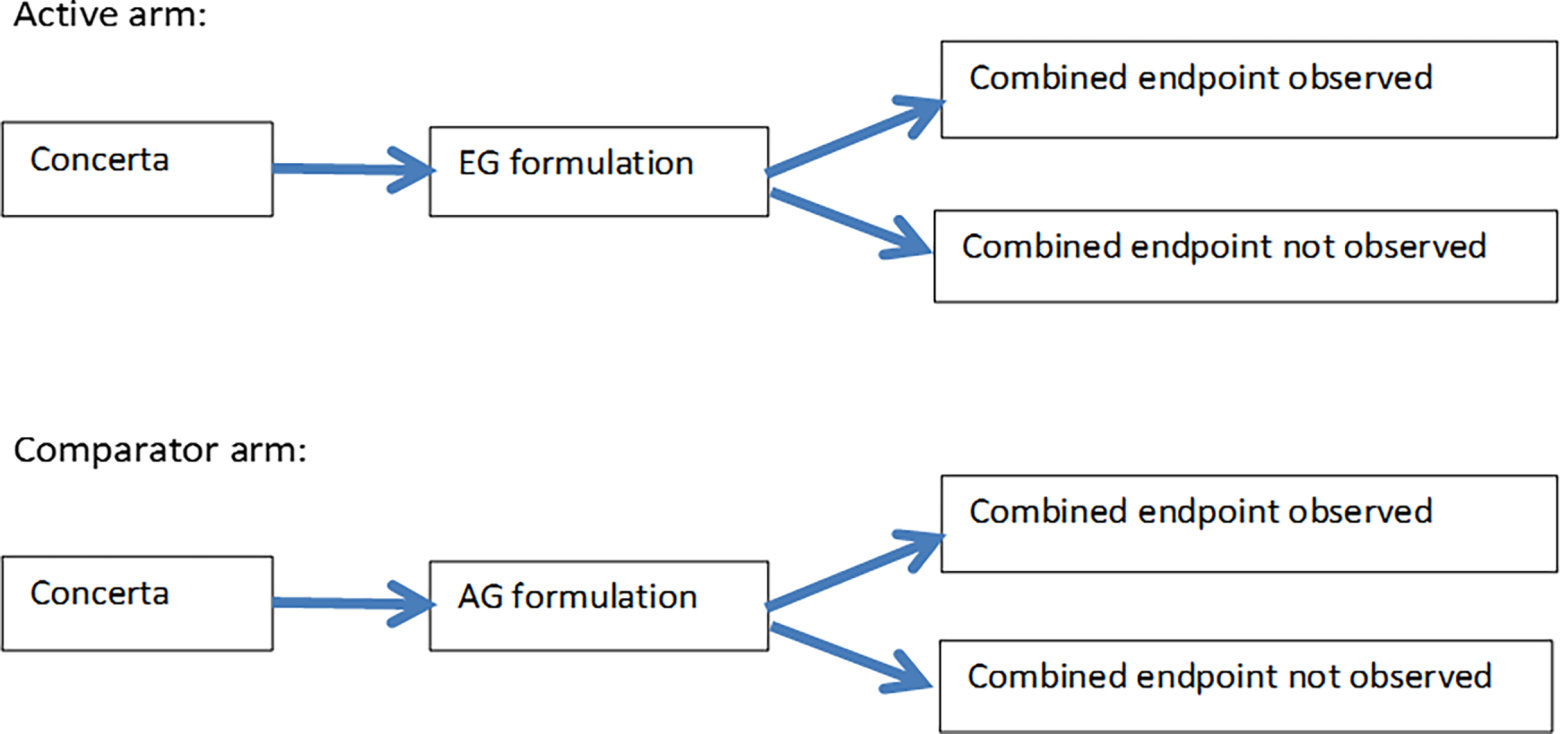
Schematic of study design.

The study’s combined endpoint consisted of four simple actions that might be taken in response to the true or false perception that the pharmacologic effects of the generic formulations were not the same as those of Concerta. The references reflect the same or similar endpoints used in other studies: Switching back to Concerta, i.e., receiving a Concerta dispensing more than 3 days and less than 61 days after the index date [4, 12, 13]; changing the use of immediate release (IR) MPH, i.e., receiving it in the 60 days after the index date but not in the 60 days before, or (conversely) not receiving it in the 60 days after the index date but receiving it in the 60 days before [3, 4]; starting a different ADHD medication (receiving, after the index date, a dispensing of an active drug substance for ADHD that was not dispensed in the 60 days before the index date)–[3, 4, 14]; or discontinuing the use of LA MPH (having at least one day after the index date that was more than 15 days since the end of the days’ supply of the most recent LA MPH dispensing) [3, 14]. Fig 1 summarizes the study design.

Based on a preliminary tabulation, we established that only a modest proportion of subjects had their observation time for the primary endpoint truncated by leaving the cohort for reasons other than meeting the primary endpoint or reaching the end of the study period. We therefore adopted the risk ratio [RR] (EG arm vs. AG arm) for developing the combined endpoint as the primary outcome statistic with no adjustment for observation time. For each subject, the probability of switching fromConcerta to the AG vs. switching from Concerta to the EG was described by a propensity score using regularized logistic regression that included all diagnoses and medications and defined the variables for inclusion in the score [15, 16]. Subjects whose propensity score was in the top or bottom 5% were excluded from the propensity score adjusted analyses. We adjusted for confounding by pooling over quintiles of propensity score using logistic regression. Because logistic regression gives odds ratios (ORs) rather than RRs, the adjusted estimates are given as ORs. For the secondary endpoints, truncation of observation time by the occurrence of the primary endpoint was potentially substantial and informative. These endpoints were assessed using a risk model as above, with the understanding that this would tend to yield underestimates if, as expected, the primary endpoint occurred more often in the EG arm than in the AG arm.

The study was conducted according to a protocol registered in advance with ClinicalTrials.gov. Changes to the protocol were recorded as amendments and were made before the study outcome statistic was calculated. The changes with potential to affect the main outcome were: Restricting the index date to the period when the generic formulations were available; changing the observation period before and after the index date from 90 days to 60 days in order to increase the number of study subjects; adopting a RR as the primary outcome statistic measure rather than a statistic that adjusted for observation time; excluding the year of the index date (2014 vs. other) from the propensity score and making it a covariate—because this would improve the efficiency of the propensity score in view of the substantial difference between the index year distributions of the AG arm and EG arm; and adding two sensitivity analyses: One that excluded subjects who had had a switch to a generic LA MPH at some time prior to their index date, and one that excluded subjects whose index date was after Sept 13, 2014 so some of their observation time fell after the FDA announcement in November 2014 regarding non-equivalency of the EG formulations and Concerta.

There were two unplanned analyses. In one, the adequacy of the propensity score was assessed by means of calibration against negative controls [17]. Negative controls are outcomes that are very unlikely to be related to the exposure of interest. If, after adjustment, more of them differ across the two exposure arms than would be expected by chance, that excess is evidence of residual confounding. In the other, the year of the index date (2014 vs. other) was included in the propensity score and not treated as a covariate in the logistic analysis used to develop the adjusted OR from the estimates by quintile of propensity score.

## Results

Table 1 describes the steps by which the study identified 732 study-eligible subjects, of whom 124 switched from Concerta to the EG and 608 to the AG formulation. The larger number of subjects in the latter group may be explained by the earlier introduction of the AG formulation (May 2011) than the EG formulations (December 2012, July 2013). Table 2 describes selected baseline characteristics and the reasons for the end of cohort membership. In each cohort, approximately 14% of subjects left the cohort for reasons other than meeting the primary study endpoint or reaching the end of the observation period, and the mean and median numbers of days in the two cohorts were similar. In the EG cohort, 49 (39.5%) of 124 subjects met the primary study endpoint, and in the AG cohort 200 (32.9%) of 608 subjects did so, resulting in a crude RR of 1.20 (95% confidence interval [CI] 0.94, 1.54).

**Table 1:** Selection of the study cohort.

Criterion	Persons
Had Concerta then had EG or AG between December 2012 and December 2014	4705
Observed 183 days after June 1, 2012	3486
ADHD Diagnosis on or after January 1, 2012	2942
Had 60 or more days of Concerta exposure followed by a switch to EG or AG	1490
Index date^1^ between December 1, 2012 and December 3, 2014	1476
If age < = 20, index date between Nov 15 and April 15 to account for “methylphenidate breaks”	1075
Did not receive both EG and AG on index date	1066
Age on index date in range (6 to 65 years)	1065
No Anxiety Diagnosis any time after 6/1/2012 and before index date	900
No Bipolar Diagnosis any time after 6/1/2012 and before index date	881
No other Excluded Diagnosis^2^ any time after 6/1/2012 and before index date	865
No non-tablet methylphenidate	853
No other excluded medication^3^	732
No other exclusion criterion^4^	732
Total Subjects in the study group	732
Number in EG arm	124
Number in AG arm	608

1 Date of substitution of the EG or AG formulation for Concerta

2 Renal or hepatic insufficiency, schizophrenia, bipolar disorder or mania, glaucoma, Tourette’s syndrome, nervous tension, narrowing of esophagus, stomach or intestine, pregnancy.

3 Antidepressants, antipsychotics, medications commonly used to treat seizures or migraines, methylphenidate in any form other than a non-chewable tablet

4 Pregnancy, receipt of a Concerta dispensing with 3 days after the index date

**Table 2:** Selected characteristics of the study cohort by study arm (AG or EG).

	Switched to EG	Switched to AG
Total (N)	124	608
Sex = male (N, %)	75 (60.5%)	422 (69.4%)
Age in years (Mean, median) ^1^	26.0 (19)	20.4 (15)
Age < = 20 years (N, %)^1^	64 (51.6%)	422 (69.4%)
Number of current meds (Mean, median) ^1^	2.1 (1)	1.8 (1)
Number of ADHD meds (Mean, median) ^1^	1.1 (1)	1.1 (1)
Anxiolytic or sedative^1^ (N, %)	2 (1.6%)	5 (0.8%)
Concerta dose (Mean, median) ^1^	38.8 (36)	40.4 (36)
Using immediate release methylphenidate^1^	8 (6.5%)	38 (6.3%)
Index date in 2012 (N, %)	0 (0.0%)	139 (22.9%)
Index date in 2013 (N, %)	58 (46.8%)	417 (68.6%)
Index date in 2014 (N, %)	66 (53.2%)	52 (8.6%)
Days in cohort after index date (Mean, median)	46.6 (60)	47.2 (60)
Reason for leaving the cohort		
Met primary study endpoint	49 (39.5%)	200 (32.9%)
Left database	6 (4.8%)	62 (10.2%)
Dispensed a different LA MPH formulation^2^	10 (8.1%)	21 (3.5%)
Met an exclusion criterion other than as above	1 (0.8%)	2 (0.3%)
End of observation period	58 (46.8%)	323 (53.1%)

1 On the subject’s index date

2 Different from the LA MPH formulation (either EG or AG) to which the subject switched on the index date

Table 3 shows the standardized differences between the EG arm and the AG arm before propensity score adjustment and after propensity score adjustment for 10 selected variables. For sex = female; age < = 12 years; age 21–35 years; number of prescribed medications > median; number of ADHD medications > median; current anxiolytic, or sedative; and Concerta dose > median, the standardized differences after adjustment are either larger or only slightly smaller than the standardized differences before adjustment.

**Table 3:** Standardized differences before and after propensity score adjustment for selected characteristics of study subjects.^1^

Characteristic	EG Arm N = 124	AG Arm N = 608	Standardized Difference Before Propensity Score Adjustment	Standardized Difference After Propensity Score Adjustment
Female ^2^	0.40	0.36	0.19	0.17
Age < = 12 years ^2^	0.28	0.32	-0.09	-0.11
Age 13–20 years ^2^	0.23	0.37	-0.30	0.00
Age 21–35 years ^2^	0.20	0.15	0.13	0.14
Age > 35 years ^2^	0.28	0.15	0.32	-0.01
Number of all prescribed medications > median ^2^	0.45	0.42	0.06	-0.09
Number of all ADHD medications > median ^2^	0.01	0.01	-0.05	-0.06
Current anxiolytic or sedative ^2^	0.02	0.02	-0.03	-0.10
Concerta dose > median ^2^	0.29	0.38	-0.19	-0.19
Number of distinct procedures in 365 days before index date	9.79	8.16	0.26	0.07

1 The propensity score used regularized logistic regression that included all diagnoses and medications and defined the variables for inclusion in the score. The variables included in the table were selected based on having large standardized mean differences before adjustment.

2 Represents a binary variable

Table 4 shows for the subjects who switched to the AG formulation, and separately for those who switched to the EG formulation, the number and proportion who met the primary study endpoint and shows the primary endpoint, the adjusted OR, which was 1.23 (95% CI 0.90, 1.70). Table 5 shows the crude and adjusted ORs for the individual components of the composite endpoint and for two secondary endpoints: Having an outpatient visit for ADHD after the index date, and changing an established MPH regimen after the index date. Of the subjects in the EG arm, 24.6% and in the AG arm 19.7% switched back to Concerta within 60 days after their index date. Switching back to Concerta was the most commonly observed component of the primary endpoint: Among the 242 subjects in either arm who met the primary endpoint, 149 (61.6%) switched back to Concerta.

**Table 4:** Primary study endpoint adjusted odds ratio.

Propensity Score Quintile	Switched to EG			Switched to AG		
	Number at risk	Number meeting primary endpoint	Risk (95% CI)	Number at risk	Number meeting primary endpoint	Risk (95% CI)
Analysis pre-specified in the protocol (Year of index date excluded from the propensity score and treated as a covariate)						
**1**	24	12	0.50 (0.30–0.70)	199	62	0.31 (0.25–0.38)
**2**	36	13	0.36 (0.20–0.52)	232	72	0.31 (0.25–0.37)
**3**	11	4	0.36 (0.08–0.65)	31	8	0.26 (0.1–0.41)
**4**	27	11	0.41 (0.22–0.59)	77	29	0.38 (0.27–0.48)
**5**	20	7	0.35 (0.14–0.56)	69	24	0.35 (0.24–0.46)
**N**^**1**^	118	47	0.40 (0.31–0.49)	608	195	0.32 (0.28–0.36)
**Odds Ratio**^**2**^ **(95% CI)**	1.23 0.90–1.70) (p = 0.21)					

1 Table excludes subjects who fell in the top or bottom 5% of the propensity score distribution.

2 The summary odds ratio was calculated from the strata by logistic regression that treated year of index date as a covariate.

**Table 5:** Crude risk ratios, crude odds ratios, and adjusted odds ratios for primary endpoint, components of primary study endpoint, and two secondary endpoints.

Endpoint	Events in EG Arm (N, %^1^) Among 118 subjects^2^	Events in AG Arm (N, %^1^) Among 608 subjects^2^	Crude RR (95% CI)	Crude OR (95% CI)	Adjusted OR (95% CI)
Primary endpoint (components below)	47 (39.8%)	195 (32.1%)	1.24 (0.97–1.59)	1.40 (0.93–2.10)	1.23 (0.88–1.68)
Switched back to Concerta	29 (24.6%)	120 (19.7%)	1.25 (0.88–1.78)	1.33 (0.82–2.09)	1.22 (0.79–1.82)
Changed IR MPH use^3^	9 (7.6%)	34 (5.6%)	1.36 (0.67–2.75)	1.39 (0.62–2.92)	1.42 (0.63–2.88)
Began a new ADHD medication	5 (4.2%)	40 (6.6%)	0.64 (0.26–1.58)	0.63 (0.22–1.54)	0.76 (0.26–1.78)
Stopped Concerta and LA MPH	5 (4.2%)	18 (3.0%)	1.40 (0.53–3.70)	1.45 (0.47–3.83)	1.23 (0.40–3.14)
Secondary endpoints					
Changed an established MPH regimen	13 (11.0%)	47 (7.7%)	1.43 (0.80–2.56)	1.48 (0.75–2.78)	1.52 (0.78–2.74)
Had outpatient visits for ADHD	28 (23.7%)	175 (28.8%)	0.82 (0.58–1.16)	0.77 (0.48–1.21)	0.84 (0.55–1.24)

1 The percentages of the 118 or 608 subjects in each arm who had the endpoint. Some subjects had more than one endpoint.

2 Table excludes subjects who fell in the top or bottom 5% of the propensity score distribution. The summary odds ratio was calculated from the strata by logistic regression that treated year of index date as a covariate.

3 Subjects who were not dispensed IR MPH in the 60 days up to and including the index date, and were dispensed IR MPH in the 60 days after it or, conversely, were dispensed IR MPH in the 60 days up to and including the index date, and were not dispensed IR MPH in the 60 days after it

Examination of the negative controls did not suggest an association with study arm (AG or EG) in either the planned or the unplanned analysis. In the sensitivity analysis that excluded subjects who had a switch to the AG formulation or the EG formulation before their index date, the adjusted OR was 1.24 (95% CI 0.87, 1.72). In the sensitivity analysis that excluded subjects whose index date was after September 13, 2014, the adjusted OR was 1.23 (95% CI 0.87, 1.69). In the unplanned analysis that included the year of the index date (2014 or other) in the propensity score and did not include it as a covariate in the logistic model, the adjusted OR was 1.00 (95% CI 0.69, 1.44), and the two sensitivity analyses described above yielded adjusted OR’s of 0.96 (95% CI 0.63, 1.42), and 0.95 (95% CI 0.64, 1.37), respectively.

## Discussion

The modest difference observed in the composite endpoint between subjects who switched from Concerta to the EG formulation vs. the AG formulation of long-acting MPH was in the expected direction but did not reach statistical significance versus a null hypothesis of no difference, and there is concern about potential residual bias. Though the distribution of the negative controls did not raise concerns about confounding, the limited improvement of the standardized difference for the selected variables made it clear that the adjustment by quintile of propensity score did not adequately address all potential confounders and the unplanned estimate of the adjusted OR suggested no difference between switching to the truly equivalent AG formulation vs. switching to the nominally equivalent EG formulation. Switching back to Concerta was the most commonly observed component of the composite endpoint and has considerable face validity as a measure of a perceived difference between the Concerta and the generic formulation. The fact that in the AG arm, approximately 33% of subjects had the combined endpoint and approximately 20% switched back to Concerta was unexpected. It suggests that the non-pharmacological effects of switching from a branded formulation to a generic formulation were substantial. This is consistent with the finding in a recent review [18–20] that the mean efficacy of MPH in pediatric and adolescent ADHD may not be far above the minimum that is clinically relevant.

Our results demonstrate the importance of a general issue in the design of retrospective database studies of substituting a generic formulation of a medication for the branded formulation of the same medications when there is a subjective component to the medication’s effect. The high frequency of the study’s combined endpoint, after switching from branded Concerta to the AG formulation with identical pharmacology makes it clear that, without an appropriate comparator, such studies may be biased toward finding a difference, but the most obvious appropriate comparator, a group of patients switched to a truly equivalent generic, is not usually available.

Among the strengths of the present study was the use of a comparator consisting of subjects who switched from the branded formulation to a generic, the AG formulation, which was identical to the branded formulation except for external packaging. With this design, the subjects in the EG arm experienced both the pharmacologic effects and the non-pharmacologic effects of switching, and the subjects in the AG arm experienced only the non-pharmacologic effects of switching. However, we were not able to take full advantage of this strength of the design and do an unbiased comparison with the non-pharmacologic effects of the switch isolated from the pharmacological effects of the switch because we were not able to address adequately the other potential confounders. Other strengths included use of a large health services database to identify the population of interest, a protocol that was registered in advance, identification of all substantial changes from that protocol, use of a propensity score to adjust for multiple confounders, and use of negative controls to provide an additional check on the adequacy of the propensity score adjustment. Limitations include the modest number of subjects in the EG arm, which limited the precision of the estimate and may have contributed to the finding of no statistically significant difference between the frequency of the combined endpoint in the two study arms, limited the ability of the propensity score to adjust adequately for the many potential confounders, left residual bias in the adjusted estimate, and may have contributed to the difference between the estimates from the planned and the unplanned approach to adjusting for confounding; the absence from the propensity score of information on copayments [21]; the increased risk of confounding in prevalent user studies compared to new user studies; and the fact that the study was done in a database that represents mainly privately insured subjects so its results may not generalize to other populations. Finally, this study examined a medication whose intended effect is assessed by somewhat subjective criteria, and it is not clear to what extent the same concerns might apply to a study of branded to generic switching of a medication, such as a lipid-lowering agent, whose intended effect is measured more objectively.

## Conclusion

This study did not detect a difference between the proportion of people who met the study endpoint in the two study arms, i.e. between those who switched to a generic formulation that was identical to Concerta except for external packaging and those who switched to the comparison generics. The substantial proportion of subjects in the AG arm who met the study endpoint, implies that studies of generic substitution must address the non-pharmacologic effects of the substitution.
